# Building effective multilevel HIV prevention partnerships with Black men who have sex with men: experience from HPTN 073, a pre‐exposure prophylaxis study in three US cities

**DOI:** 10.1002/jia2.25180

**Published:** 2018-10-18

**Authors:** Darrell P Wheeler, Jonathan Lucas, Leo Wilton, LaRon E Nelson, Christopher Hucks‐Ortiz, C Chauncey Watson, Craig Hutchinson, Kenneth H Mayer, Irene Kuo, Manya Magnus, Geetha Beauchamp, Steven Shoptaw, Lynda Marie Emel, Ying Q Chen, Lisa Hightow‐Weidman, Sheldon D Fields

**Affiliations:** ^1^ Iona College New Rochelle NY USA; ^2^ Science Facilitation Department FHI 360 Durham NC USA; ^3^ Department of Human Development State University of New York at Binghamton Binghamton NY USA; ^4^ Faculty of Humanities University of Johannesburg Johannesburg South Africa; ^5^ School of Nursing University of Rochester Rochester NY USA; ^6^ Centre for Urban Health Solutions Li Ka Shing Knowledge Institute St. Michael's Hospital Toronto ON Canada; ^7^ John Wesley Community Health Institute, Inc. Commerce CA USA; ^8^ Gilead Sciences‐333 Velocity Way Foster City CA USA; ^9^ Westside Community Mental Health Center San Francisco CA USA; ^10^ The Fenway Institute Fenway Health Boston MA USA; ^11^ Harvard Medical School Boston MA USA; ^12^ Beth Israel Deaconess Medical Center Boston MA USA; ^13^ Department of Epidemiology and Biostatistics Milken Institute School of Public Health George Washington University Washington DC USA; ^14^ Statistical Center for HIV/AIDS Research & Prevention (SCHARP) Vaccine and Infectious Disease Division (VIDD) Fred Hutchinson Cancer Research Center Seattle WA USA; ^15^ Department of Family Medicine David Geffen School of Medicine University of California Los Angeles (UCLA) Los Angeles CA USA; ^16^ Fred Hutchinson Cancer Research Center Seattle WA USA; ^17^ Division of Infectious Diseases University of North Carolina at Chapel Hill Chapel Hill NC USA; ^18^ School of Health Professions New York Institute of Technology Northern Boulevard NY USA

**Keywords:** African American, homosexual, community engagement, PrEP research

The sub‐population at greatest risk for HIV infection in the United States is Black men who have sex with men (BMSM), and there is an urgent need for effective HIV prevention interventions among them 1. Despite advances in biomedical and behavioural interventions, healthcare systems continue to fail to slow the epidemic among BMSM. This is particularly the case among young men 2, who have an estimated lifetime risk of HIV infection of up to 50% 3. It has been well demonstrated that tenfovir disiproxil fumarate (TDF) and emtricitabine (FTC) as pre‐exposure prophylaxis (PrEP) are effective in protecting those at risk of HIV acquisition from sex or injection drug 4, 5, but prescriptions to BMSM have sorely lagged behind other affected populations, including gay and bisexual White men 5, 6.

Myriad structural characteristics, including poor health, poverty, stigma, high rates of incarceration, inadequate housing, lack of health insurance, decreased educational attainment and unemployment, impede recruitment and retention of BMSM in studies 6, 7. This constellation of factors is likely responsible for both high HIV prevalence and low uptake of PrEP (and other interventions) 8. Clearly, effective interventions to increase PrEP uptake among BMSM are urgently needed; yet, in public health and medicine, we continue to miss the mark 5.

Just one demonstration project cannot overcome this full range of barriers. However, in the HIV Prevention Trials Network (HPTN) 073 study discussed in this view point article, we focused on what we perceived to be primary factors precluding adequate study of HIV prevention interventions among BMSM: lack of indigenous scientific leadership evident throughout the HIV prevention research field; and low BMSM enrolment in nearly all PrEP studies, including the initial iPrEX trial itself 2, 9. The key to our approach was purposefully increasing representation of Black researchers in leadership roles 10. HPTN 073 was unique in its being led by researchers of the community under study and its thoughtful connectivity and engagement with this community at all points of study design, implementation, analysis and dissemination. This approach was grounded in the foundational work of Andrasik *et al*. 11, who identified three key themes as barriers to engage BMSM in HIV prevention research: authentic/true partnerships with community‐based organizations; a real investment in the Black gay community; and the follow‐up to truly inform and educate the community after the study is completed.

HPTN 073 was an open‐label antiretroviral PrEP demonstration project. Eligible BMSM aged 18 years and older in three US cities were offered daily oral co‐formulated FTC/TDF, with primary outcomes being PrEP uptake/initiation and adherence 12. The majority of the HPTN 073 leadership team, including the protocol chair and co‐chair, behavioural scientist, intervention developer and key staff members, worked together on a previous BMSM study (HPTN 061) 7, 13, 14 and had many years of significant linkages to BMSM communities and organizations across the US. In addition, along with key staff at all sites, they were also members of the BMSM community.

The behavioural intervention developed for this study was client‐centred care coordination (C4), which incorporated theoretical and public health approaches from comprehensive risk counselling and self‐determination theory 15 tailored specifically for BMSM supporting participants’ evaluation of their risk for HIV and personal ability to accept and adhere to PrEP if they elected to take it 16, 17. The study sites included Washington, District of Columbia; Los Angeles, California; and Chapel Hill, North Carolina.

HPTN 073 included standardized and rigorously evaluated site development activities to assist study teams in assessing and enhancing their readiness to work with BMSM, including a comprehensive cultural competency component designed by the HPTN Black Caucus [presence/place at the table (PAT)], specifically manualized for the study. Intrinsic to this approach was the concept that the study itself was not objectifying disconnected community members, but rather partnering members of the community with esteemed academic experts. This allowed participants to realize from the outset that the results of this study were intended to impact the lives of men in their communities in real time. This comprehensive approach embraced all facets of study, allowing brisk recruitment, strong retention and collection of high‐quality data. In addition, emerging from this demonstration project are new scalable approaches for engaging historically under‐represented researchers in leadership roles in the future.

HPTN 073 sites successfully recruited and screened 344 people and ultimately enrolled 226 BMSM between February 2013 and September 2014, retaining 92% of participants for the 12‐month follow‐up]. The findings from the HPTN 073 study suggest that behavioural and biomedical interventions can be used in combination to support BMSM acceptance of, adherence to and benefit from oral PrEP 16, 17.

It is already known that PrEP works when taken and that removing barriers helps uptake. This study went further showing the critical importance of meaningful engagement between the community and researchers who embody the priorities of the participants. BMSM leaders of the study ensured that all facets of HPTN 073 were rigorously performed to support the needs of the BMSM themselves, and not just to prepare an article read only by researchers. The depth of community support, from recruitment to evaluation and analysis, were consistent in all sites. Study participants and community members identified the significance of having BMSM leadership. By supporting staff's awareness of and ability to engage in active listening, critical examination of barriers to service delivery and attention to understanding multilevel needs of BMSM, the HPTN 073 staff created supportive environments in which men could develop HIV prevention approaches, including PrEP tailored to their needs.

The researchers used a culturally tailored PrEP programme for BMSM with intentional indigenous scientific leadership and ongoing codified efforts to ensure adequate training and cultural competency; this led to numerous positive outcomes 12. The role of knowledge in the form of BMSM leadership is a key factor in supporting future research efforts. Those in control of access to, and interpretation of knowledge and research processes can and do shape what is validated and what is not. As Tunde Wey writes, “It is about who gets to create us and what those representations mean for our lives… The world has a way of turning on the careless words of fools.” (2018, March 11, SF Chronicle) 18.

## Competing interests

The authors declare that they have no competing interests.

## Authors’ contributions

All authors have contributed substantially to the conception and design of the manuscript or acquisition, analysis or interpretation of the data for this work. Each author has participated in the drafting or revision of the content. Additionally, DPW was the chair of the HPTN 073 study and SDF was the co‐chair of the study. LW was the lead behavioural scientist and LEN was the lead implementation scientist for the study. JPL provided scientific leadership in all phases of community engagement for the study. MM, LHW and SS were the site investigators for the study in their respective cities. KHM, DPW, SDF, LW, LEN, MM, LHW and SS provided scientific leadership in the conceptualization, development and implementation of the study. GB and YQC provided statistical analysis for the study. LME supervised data management for the study. KHM served as a protocol team member and provided scientific‐, medical‐ and health‐related expertise for the study. IK provided research, data analysis and interpretation for the study and was also a co‐PI for her respective site. CCW served as the chair of the HPTN Black Caucus. CHO and CH served as HPTN Black Caucus Vice Chairs and provided socio‐cultural expertise for the study. All authors contributed to the writing of the manuscript. All authors have read and approved the final manuscript.
